# Enhanced Lithium-Ion Transport in Lithium Metal Batteries Using ZSM-5 Nanosheets Hybridized Solid Polymer Electrolytes

**DOI:** 10.3390/polym16111604

**Published:** 2024-06-05

**Authors:** Xiaoyan Hu, Jialiang Liu, Baoquan Zhang

**Affiliations:** State Key Laboratory of Chemical Engineering, School of Chemical Engineering and Technology, Tianjin University, 135 Ya Guan Road, Jinnan District, Tianjin 300350, China; huxy1108@tju.edu.cn (X.H.); jialiang@epri.sgcc.com.cn (J.L.)

**Keywords:** solid polymer electrolyte, ZSM-5 nanosheet, organic–inorganic composite electrolyte, lithium metal battery

## Abstract

Solid polymer electrolytes (SPEs) are the key components of lithium metal batteries to overcome the obstacle of insecurity in conventional liquid electrolytes; however, the trade-off between their ionic conductivity and mechanical properties remains a significant challenge. In this work, two-dimensional ZSM-5 nanosheets as fillers are incorporated into a poly(ethylene oxide) (PEO) matrix and lithium salts to obtain composite polymer electrolytes (CPEs). The improved physicochemical and electrochemical properties of the CPE membranes are characterized in full detail. Stripping/plating measurements in symmetric Li/Li cells and cyclic charge/discharge tests are performed to investigate the cyclability and stability of the CPEs. All-solid-state LiFePO_4_/Li batteries deliver excellent cycling performance with an initial discharge capacity of 152.3 mAh g^−1^ and 91.4% capacity retention after 200 cycles at 0.2 C, with a discharge specific capacity of 118.8 mAh g^−1^ remaining after 350 cycles at 0.5 C. Therefore, CPEs containing ZSM-5 nanosheets are a promising option for all-solid-state lithium-ion batteries.

## 1. Introduction

With the fast-growing development of electric vehicles, efficient and safe energy storage devices are highly demanded. In consideration of their high energy density and safety, lithium-ion batteries (LIBs) have dominated the market of next-generation storage devices [1,2]. Due to the inherent flammability of conventional organic electrolytes, LIBs suffer from severe safety threats. It has been found that non-volatile and non-flammable solid polymer electrolytes (SPEs) could substantially reduce this fire risk [3]. Obviously, their inherent flexibility, lightweight nature, low cost, and facile processability make SPEs a promising electrolyte for LIBs. Up until now, SPEs have achieved commercial-scale demonstration in the Li-ion battery industry. Poly(ethylene oxide) (PEO) has attracted great interest owing to its ability to dissolve lithium salts and conduct Li ions, demonstrating great potential in SPEs as a polymer matrix [4]. Limited by their inherent semicrystalline structure, Li ions can only transfer in the amorphous regions of PEO matrices by ion hopping and according to the segmental motion of polymer chains. This leads to low ionic conductivity at room temperature and poor mechanical properties, which inevitably hinder their further commercialization. Hence, PEO-based SPEs with superior ionic conductivity and high mechanical strength are urgently required [5].

At present, several strategies have been proposed, including (but not limited to) blending high-modulus polymers [6,7,8] such as poly(vinylidene fluoride) (PVDF), polyimide (PI), polyacrylonitrile (PAN), polymethyl methacrylate (PMMA), and polyethylene (PE). These rigid polymers might effectively improve the mechanical strength. Thus, the electrostatic action between the filled polymers and PEO segments would reduce the crystallization tendency of PEO to improve the ion conductivity. Furthermore, hybrid inorganic nanoparticles are considered another promising candidate. Up to now, many inorganic nanofillers have been introduced into SPEs [9,10], including inorganic oxides, polyhedral oligomeric silsesquioxanes (POSS), metal–organic frameworks (MOFs), and active fillers like NASICON, garnet, etc. Incorporated inorganic nanoparticles can increase the amorphous phase of the PEO polymer host, derived from the Lewis acid interactions between the fillers and lithium salt. More free lithium ions dissolved from the lithium salt and a higher ionic conductivity would be expected.

Among inorganic nanoparticles, zeolites are crystalline aluminosilicates with a molecular sieving function, adsorption capacity, and excellent physicochemical stabilities. They have been extensively applied in the petrochemical industry. In recent decades, our research group has utilized zeolites for the molecular separation of gas mixtures [11,12] and liquid solutions [13,14]. Owing to the outstanding characteristics of zeolites, they have been extensively investigated for enhancing the ionic conductivity and mechanical strength of organic–inorganic composite polymer electrolytes (CPEs) [15]. With an ultrahigh surface area and ordered nanosized pores, mesoporous silica is conducive to absorbing and confining plasticizers, providing large-scale nanochannels for ion transport. Apart from inhibiting the crystallization tendency of PEO to increase the amorphous regions, a rapid Li-ion transport pathway between zeolitic fillers and the PEO matrix can greatly improve the electrochemical stability and mechanical strength of SPEs. Li et al. [16] employed SSZ-13 as a highly ordered porous additive to produce PEO-based electrolytes, which significantly improved the Li^+^ conductivity (1.91 × 10^−3^ S cm^−1^ at 60 °C) and widened the electrochemical stability window to 4.7 V vs. Li^+^/Li. Xi et al. [17,18] found that ZSM-5 could obviously enhance the ionic conductivity and other electrochemical properties of PEO-based polymer electrolytes. Through the regular replacement of [AlO_4_]^–^ with [SiO_4_] in the framework of microporous molecular sieves, the cation exchange centers facilitated the exchange of Li ions into the well-defined channels for charge balance. Zhang et al. [19] prepared a nanocomposite electrolyte using the method of electrospinning with subsequent thermal cross-linking. Firmly attached to the membrane through chemical bonds, the ZSM-5 molecular sieves contributed to enhancing the electrolyte uptake, thermal stability, and ionic conductivity. However, the excessive doping amount caused inhomogeneous dispersion in the polymer matrix.

To address the above challenge, a large specific surface area with an ultrathin structure should be conducive to improving the Lewis acid interactions between two-dimensional (2D) fillers and a polymer matrix [20,21,22]. So far, the use of 2D zeolite nanosheets as an additive for SPEs has not been publicly reported. In this work, we constructed a novel CPE by introducing inorganic ZSM-5 nanosheets into a PEO polymer matrix. The ZSM-5 nanosheet fillers significantly reduced the crystallinity of the PEO. The strong Lewis acid interactions of the hydroxyl on the surface-coordinated unsaturated Al atoms in the ZSM-5 nanosheet skeleton and the porous electronegativity channels not only promoted the dissociation of lithium salt but provided new, fast conductive pathways for Li^+^ as well. The Li^+^ transference number and the mechanical strength of the SPE could be efficiently improved. The enhanced comprehensive performance of CPEs with ZSM-5 nanosheets make them a promising candidate for next-generation lithium metal batteries.

## 2. Experimental Sections

### 2.1. Materials and Chemicals

Tetraethyl orthosilicate (TEOS, 98%) and tetrapropylammonium hydroxide (TPAOH, 40%) were obtained from Sigma-Aldrich (St. Louis, MO, USA). 2-butanone (99.5%) and ethyl acetate (99.5%) were obtained from Tianjin Yuanli Chemical Co., Ltd. (Tianjin, China). 1,5-diaminopentane (98%) was purchased from Shanghai Taitan Technology Co., Ltd. (Shanghai, China). 1-iodopropane (99%) was obtained from Shanghai Meryer Chemical Co., Ltd. (Shanghai, China). PEO (Mn = 5 × 10^5^ g/mol) and bis (trifluoromethane) sulfonimide lithium (LiTFSI, 99.9%) were obtained from Heowns Co., Ltd. (Tianjin, China). Acetonitrile (CH_3_CN, AR grade) and N-methyl-2-pyrrolidone (NMP, AR grade) were purchased from Aladdin Chemical Co., Ltd. (Shanghai, China). LiFePO_4_ (LFP) and carbon black (super P) were supplied by Candlelight New Energy Technology Co., Ltd. (Dongguan, China). Lithium foil was received from Tianjin China Energy Lithium Co., Ltd. (Tianjin, China). All the reagents were used as received without further purification. Deionized water was utilized throughout the experiments.

### 2.2. Synthesis of ZSM-5 Nanosheets

The ZSM-5 nanosheets were synthesized by using the seed-mediated growth method [11,23]. Firstly, silicalite-1 crystals were prepared according to a hydrothermal growth process. A precursor sol with a molar composition of 25 TEOS:9 TPAOH:480 H_2_O was crystallized in a 50 °C oven for 6 days. The resulting product was filtered with a 0.45 μm polypropylene syringe filter, and then the filtrate was heated in an oven at 100 °C for 3 days, with ~30 nm silica-1 crystals collected by centrifugation. Bis-1,5(tripropyl ammonium) pentamethylene diiodide (dC5) was synthesized according to the alkylation of 1,5-diaminopentane with 1-iodopropane. Using dC5 as the structure-directing agent (SDA), ZSM-5 nanosheets were obtained according to the secondary growth of silicalite-1 seeds in a precursor sol with molar composition of 80 TEOS:3.75 dC5:20 KOH:9500 H_2_O, at 140 °C for 4 days. The synthesized ZSM-5 nanosheets of a uniform thickness were separated by sonication and centrifugation.

### 2.3. Preparation of Electrolyte Membranes

The electrolyte membranes were prepared using the conventional solvent casting technique. The Li:EO ratio was controlled at 1:13. First, 0.375 g of LiTFSI was dissolved in 15 mL of acetonitrile to form a homogeneous solution, in which the ZSM-5 nanosheets were subsequently dispersed. The used weight percentages of the ZSM-5 nanosheets were 0.5%, 1.0%, and 1.5%, respectively. Afterwards, 0.75 g of PEO powder was added into the above suspension and stirred for 12 h to obtain the final casting solution, which was poured into a Teflon plate and dried at ambient temperature. The residual solvent was completely removed in a vacuum oven at 60 °C for 24 h. The standard PEO/LiTFSI SPE membrane was prepared using the same method for comparison. All the electrolyte samples were stored in an argon-filled glove box before use.

### 2.4. Preparation of LFP Cathode

The LFP working electrode consisted of 80% LFP cathode active material, 10% super P, and 10% PEO as binder. To prepare a slurry, 8 g of LFP, 1 g of PEO, and 1 g of super P powder were dispersed in 15 mL NMP, which was blade-coated onto carbon-coated aluminum foil and dried in a vacuum oven at 60 °C for 24 h until the residual NMP was completely eliminated. The electrodes were punched into 16 mm diameter disks and stored in an argon-filled glove box for battery assembly.

### 2.5. Characterization

The surface morphologies of the ZSM-5 nanosheets and electrolyte films were measured using a field emission scanning electron microscope (SEM, S4800, Hitachi, Tokyo, Japan). X-ray diffraction (XRD, D8 FOCUS, Bruker, MA, USA) was used to study the crystallinity of the ZSM-5 nanosheets and electrolyte samples. The crystalline melting point (T_m_) of the SPE and CPEs was determined using differential scanning calorimetry (DSC, DSC 200F3, Netzsch, Selb, Germany). The samples were scanned from 30 to 80 °C to detect endothermic reactions with a heating rate of 10 °C min^−1^. The mechanical strength of the samples was characterized using nanoindentation tests (NanoTest Vantage, MML, London, UK). A rigid cylinder probe loaded force onto a sample until 1 mN at ambient temperature. Under a nitrogen atmosphere, a thermogravimetric analysis (TGA, TGA5500, TA, New Castle, DE, USA) was performed in a temperature range from 30 to 600 °C with a heating rate of 10 °C min^−1^.

### 2.6. Electrochemical Test

The electrolyte films were sandwiched between two stainless steel blocking electrodes in a CR2032 coin cell, assembled in a glove box filled with pure argon. Electrochemical impedance spectroscopy (EIS) was carried out using an Autolab PGSTAT302N (Metrohm, Herisau, Switzerland). The following equation was used to calculate the ionic conductivity (σ) of the SPE and CPEs:

(1) σ=d/RS

where *d* represents the thickness (cm) of the electrolyte membrane, and *S* is the contact area between the electrolyte membrane and the stainless steel electrode. The resistance of the electrolytes was *R*, which was measured using electrochemical impedance at the open-circuit voltage in the temperature range of 20 to 60 °C. The frequency ranged from 10^6^ Hz to 10^−1^ Hz with an amplitude of 5 mV.

To test the electrochemical windows of the electrolytes, the linear sweep voltammetry (LSV) technique was performed on the Autolab PGSTAT302N electrochemical workstation with a scanning rate of 1 mV s^−1^ at 60 °C. The Li-ion transference number (t_Li_^+^) was measured using chronoamperometry and AC impedance spectra. t_Li_^+^ was calculated using

(2) $$t_{{\mathrm{Li}}^{+}}=Is/Io\;\left(\Delta V-IoRo\right)/\left(\Delta V-IsRs\right)$$

For symmetric Li/Li cells, the initial current *I_o_* and the steady-state current *I_s_* were tested by DC polarization with a voltage pulse (Δ*V*) of 10 mV. *R_o_* and *R_s_* are the initial and steady-state resistances.

The galvanostatic cycling performance of the Li/Li symmetric cells was tested at a current density of 0.1 mA cm^−2^ and a capacity density of 0.1 mAh cm^−2^ at 60 °C. The obtained cathode electrode was employed to assemble the LFP/electrolytes/Li cells. The galvanostatic charge/discharge measurements were performed in a potential range from 2.5 V to 3.8 V (vs. Li/Li^+^) at different rates with a multichannel battery testing system (LAND CT2001A, Lanhe, Wuhan, China) at 60 °C.

## 3. Results and Discussion

### 3.1. Characterization and Performance of ZSM-5 Nanosheets and CPE Membranes

**Figure 1:**
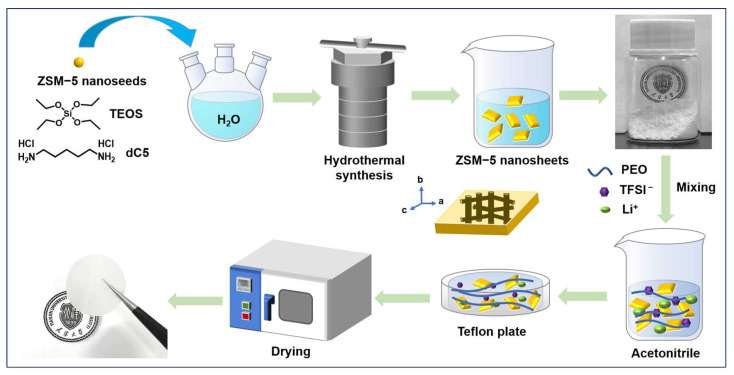
The synthetic of CPEs with ZSM-5 nanosheets.

Figure 1 shows a schematic picture of the synthesis procedure for ZSM-5 nanosheets and CPEs with ZSM-5 nanosheets. The ZSM-5 nanosheets were prepared following the procedure in our previous report [11]. The prepared ZSM-5 nanosheets are white powder. Simultaneously, an image of the as-prepared CPEs with ZSM-5 nanosheets is displayed on top of a badge of Tianjin University.

**Figure 2:**
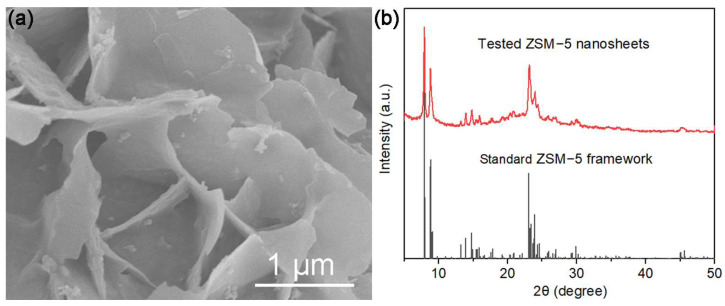
(**a**) SEM image and (**b**) XRD pattern for ZSM-5 nanosheets.

The following, Figure 2, shows the morphologies and crystal structures of the synthetic ZSM-5 nanosheets. The ZSM-5 nanosheets exhibit irregular morphologies with small lateral dimensions. Therefore, the flaky morphology of the ZSM-5 nanosheets is consistent with that in a previous literature report [23]. The XRD analysis reveals the characteristic peaks of the ZSM-5 nanosheets and the standard ZSM-5 framework, indicating the synthesized ZSM-5 nanosheets are identical to the standard ZSM-5 framework in crystal structure (Figure 2b).

**Figure 3:**
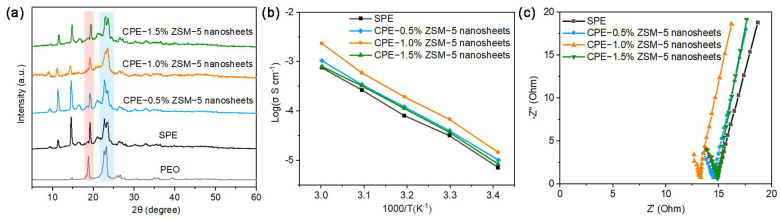
(**a**) XRD patterns of SPEs and CPEs with ZSM-5 nanosheets; (**b**) comparative Arrhenius plots; (**c**) Nyquist plots at 60 °C.

The crystalline degree of the electrolytes is displayed in Figure 3a. The sharp diffraction peaks at 19° and 23° correspond to the semi-crystalline nature of PEO at room temperature. In contrast, the diffraction peak intensities of the SPEs and CPEs with ZSM-5 nanosheets decrease significantly, indicating that the introduction of LiTFSI and ZSM-5 nanosheets can effectively obstruct the PEO’s crystallization behavior by breaking the orderly arrangement of the PEO chain segments. The increasing amorphous regions in the electrolytes would imply that more flexible polymer chains should be conducive to Li ion transport, which produces a positive effect in terms of an improvement in ionic conductivity.

To confirm the effect of the ZSM-5 nanosheet fillers on the ionic conductivity of the electrolytes, Arrhenius plots of the ionic conductivity are presented from 20 to 60 °C (Figure 3b). The ionic conductivities clearly increase at first and then decrease on increasing the ZSM-5 nanosheet doping amount. Across the full temperature range, the CPE-1.0% ZSM-5 nanosheets show the highest ionic conductivity (1.46 × 10^−5^ S cm^−1^ at 20 °C, 1.34 × 10^−3^ S cm^−1^ at 60 °C), while the ionic conductivity of the blank PEO/LiTFSI electrolyte is only 4.7 × 10^−6^ S cm^−1^ at 20 °C.

Nyquist plots of the SPEs and CPEs with 0.5%, 1.0%, and 1.5% ZSM-5 nanosheets further confirm that the CPE-1.0% ZSM-5 nanosheets possess the minimum impedance value at 60 °C (Figure 3c). Therefore, the optimum ZSM-5 nanosheet content for CPEs is ca. 1.0% under different temperatures and well fitted to the Arrhenius equation. Owing to the electronegativity of the ZSM-5 nanosheet skeleton, lithium ions are absorbed onto the surface of the nanopores, enriching a continuous, fast conduction pathway for Li ions, which could promote the ionic conductivity of CPEs with ZSM-5 nanosheets.

**Figure 4:**
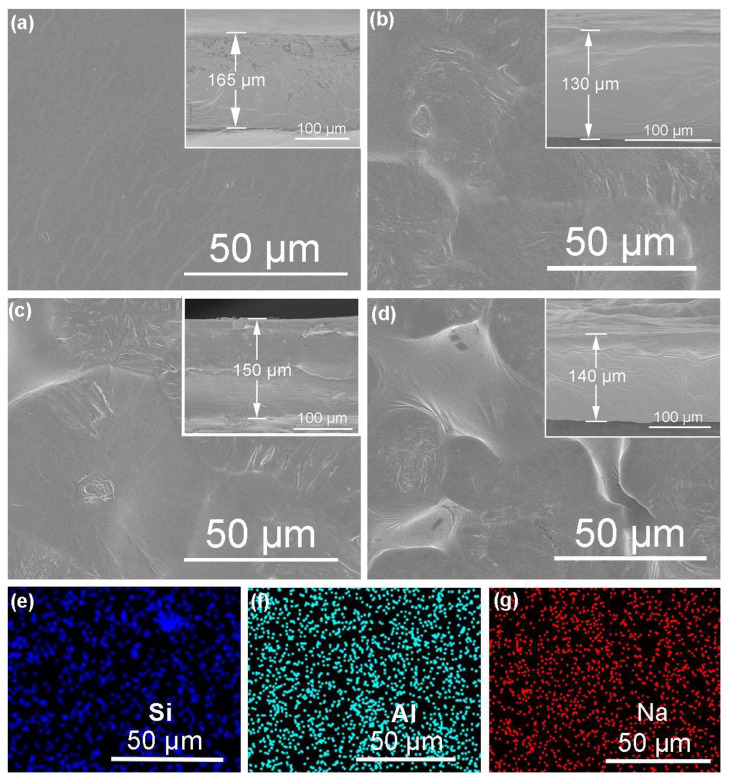
Surface and cross-sectional SEM images of (**a**) SPE, (**b**) CPE-0.5%, (**c**) CPE-1.0%, and (**d**) CPE-1.5% ZSM-5 nanosheets; EDS mapping of silicon (**e**), aluminum, (**f**) and sodium (**g**) for sample CPE-1.0% ZSM-5 nanosheet.

Micro-illustrations and cross-sections of the electrolyte films are shown in Figure 4a–d. The pure PEO-based polymer electrolyte without fillers is quite flat. When the 0.5% and 1.0% ZSM-5 nanosheet fillers are evenly dispersed, the slight phase separation is beneficial in improving the Li-ion migration rate in the PEO matrix. This is mainly attributed to the introduction of fillers with decreased PEO crystallinity. The firm adhesion on a flat surface contributes to a decrease in the interfacial impedance between the electrolyte and the electrode. However, due to the excessive uneven distribution of the 1.5% ZSM-5 nanosheet filler, rough and uneven surfaces result in increased interfacial resistance, hindering the rapid lithium-ion migration. Therefore, 1.0% is adopted as the optimized dose after comprehensive considerations. The thickness of the as-prepared electrolyte films is 130–165 µm. Additionally, the uniform distribution of Si, Al, and Na elements further confirms that the 1.0% ZSM-5 nanosheet is uniformly distributed in the PEO-based electrolyte, as shown in Figure 4e–g.

### 3.2. Electrochemical Performance of Electrolyte Membranes

**Figure 5:**
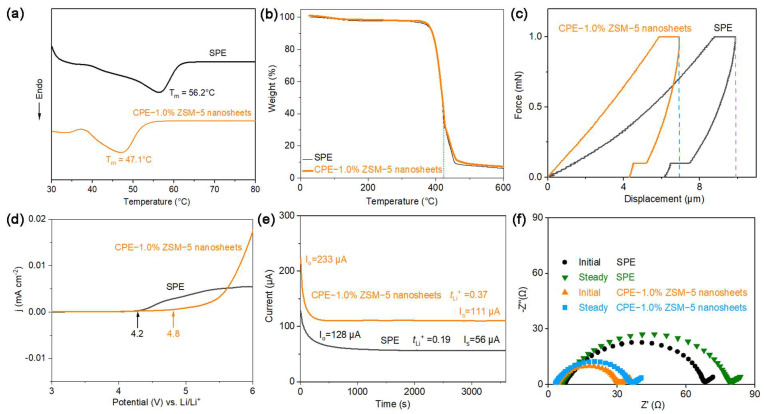
(**a**) DSC profiles; (**b**) TGA data; (**c**) nano-indentation results; (**d**) LSV curves; (**e**) chronoamperometry curves; (**f**) Nyquist plots before and after polarization.

The thermal properties of the SPE and CPE-1.0% ZSM-5 nanosheet membranes are characterized by using DSC under a N_2_ atmosphere. It can be found in Figure 5a that the T_m_ of the CPE-1.0% ZSM-5 nanosheets is 47.1 °C, lower than that of the SPE (56.2 °C). This further verifies that ZSM-5 nanosheet incorporation is conducive to decreasing the crystallinity of PEO, promoting lithium-ion conduction.

The TGA analysis of the SPE and CPE-1.0% ZSM-5 nanosheets is depicted in the temperature range from 30 to 600 °C (Figure 5b). The weight loss occurring at 370 °C is related to the decomposition of the PEO chains and lithium salts. The remaining weight at 600 °C is related to a trace amount of carbon and the ZSM-5 nanosheets, which take up about 5% of the original sample. The result demonstrates the higher thermal stability of the CPE-1.0% ZSM-5 nanosheet film.

As shown in Figure 5c, nanoindentation measurements are conducted on the electrolytes, simulating the suppression of Li dendrites. Compared with the pristine SPE, the shorter displacement corresponds to the greater hardness of the CPE-1.0% ZSM-5 nanosheets. This reveals that the superior rigidity and stability of the ZSM-5 nanosheets tend to suppress dendrite growth.

To investigate the electrochemical windows of the electrolyte membranes, LSV measurements are performed on the Li/electrolyte/stainless steel batteries at 60 °C (Figure 5d). The CPE-1.0% ZSM-5 nanosheets exhibit oxidative stability up to 4.8 V at 60 °C, higher than 4.2 V for the pure SPE. The wide electrochemical window indicates that CPE-1.0% ZSM-5 nanosheets with high electrochemical stability and feasibility would be potentially applicable to high-voltage battery systems.

The lithium-ion transference number (t_Li_^+^) is another major characteristic of electrolytes. The chronoamperometry–AC impedance method is performed to measure the t_Li_^+^ of the SPE and CPE-1.0% ZSM-5 nanosheets in the assembled symmetric Li/Li batteries (Figure 5e,f). The CPE-1.0% ZSM-5 nanosheets represent a higher t_Li_^+^ of 0.37 than that of the SPE–nanosheets (0.19). The higher Li^+^ transference number is attributed to the strong Lewis acid centers of the silicon hydroxyl groups on the surface of the porous ZSM-5 nanosheets enhancing the dissociation of the lithium salts, having a positive impact on the transport of the lithium ions.

**Figure 6:**
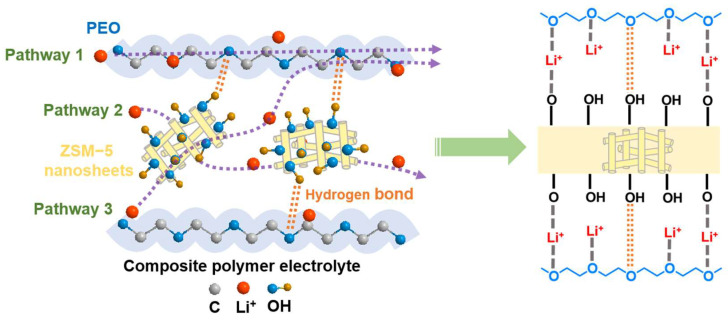
Schematic mechanism diagram of Li-ion transport pathways and Li-ion interactions between PEO and ZSM-5 nanosheets.

Figure 6 displays the Li^+^ transport pathways with their mechanism in the CPE-1.0% ZSM-5 nanosheets. The ZSM-5 nanosheet additives are uniformly encapsulated in the PEO matrix, exerting a plasticizing effect. Compared with the blank SPE, the CPE-1.0% ZSM-5 nanosheets present lower crystallinity, which is conducive to rapid lithium-ion migration. The first pathway displays the improved Li^+^ transport due to the irregular arrangement of the PEO segments. Additionally, the strong electrostatic interaction between the lithium-ions and the abundant electronegative channels of the ZSM-5 nanosheets may provide a new lithium-ion conduction pathway (pathway 2). Furthermore, the ZSM-5 nanosheets effectively promote the dissociation of the lithium salts owing to the strong Lewis acid–base reactions of the silicon hydroxyl groups on the unsaturated Al^3+^ centers, releasing a large amount of free Li^+^. The greater Li^+^ transference number results in faster Li^+^ transport, forming another Li^+^ transport pathway (pathway 3) [24]. The hydrogen bonding interaction between the PEO chains and the hydroxyl groups may facilitate the mechanical stability of the CPEs. Consequently, the doped ZSM-5 nanosheets enhance the excellent electrochemical stability and high energy density of the lithium-ion batteries.

### 3.3. Performance of All-Solid-State Cells

**Figure 7:**
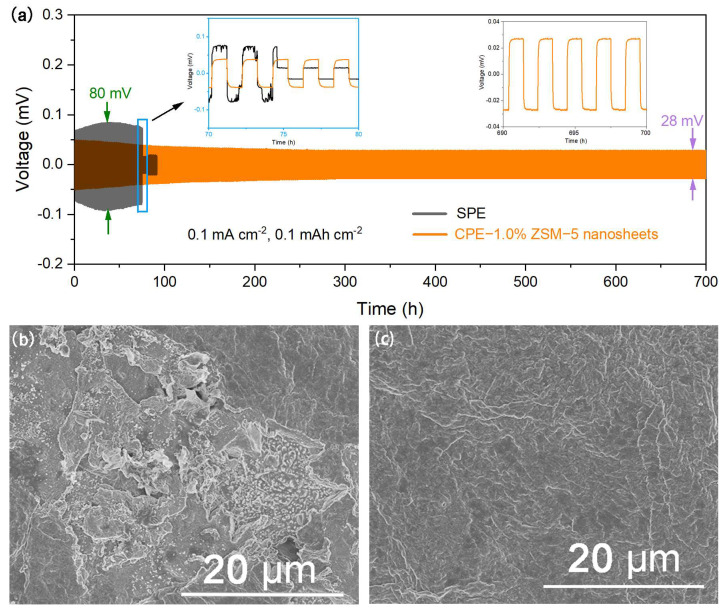
(**a**) Galvanostatic cycling performance of symmetric Li/Li batteries; the surface morphologies of Li electrodes after cycling for 50 h using SPE (**b**) and CPE-1.0% ZSM-5 nanosheets (**c**).

The long-term cycling stability of the Li electrodes at a current density of 0.1 mA cm^−2^ is presented in Figure 7a. The plating/stripping test in symmetric Li/Li cells reveals their outstanding stability for 700 h, with a stable overpotential of 28 mV, demonstrating the effective suppression of dendrites. In contrast, a short circuit occurs after only 75 h for the cell using the SPE, with a higher polarization voltage of 80 mV, which is mainly attributed to the growth of lithium dendrites derived from the inhomogeneous lithium deposition on the surface of the Li electrode. A symmetric Li/Li cell with CPE-1.0% ZSM-5 nanosheets has a smaller polarization voltage than that of a cell with an SPE. The polarization voltage decreases with the generation of a stable interface layer. This means that the interfacial impedance decreases. The rigidity of the ZSM-5 nanosheets becomes a great barrier to the growth of lithium dendrites. Moreover, better long-term cycling stability is ascribed to the higher ionic conductivity and faster Li^+^ migration for the CPE-1.0% ZSM-5 nanosheets.

In a symmetric Li/Li cell using a PEO/LiTFSI electrolyte, the inhomogeneous and aggregated Li deposition produces ample dead Li and Li dendrites on the surface of the lithium anode. Serious lithium dendrite growth and adverse interfacial reactions result in a rapid short circuit. This phenomenon is confirmed in Figure 7b. Comparatively, a stable passivation layer is formed by the uniform lithium-ion distribution on the surface of the Li anode in symmetric cells assembled with CPE-1.0% ZSM-5 nanosheets. The smooth surface topography of the Li anode can be observed after cycling for 50 h in Figure 7c. Hence, the CPE-1.0% ZSM-5 nanosheets show preferable interfacial and electrochemical stabilities.

**Figure 8:**
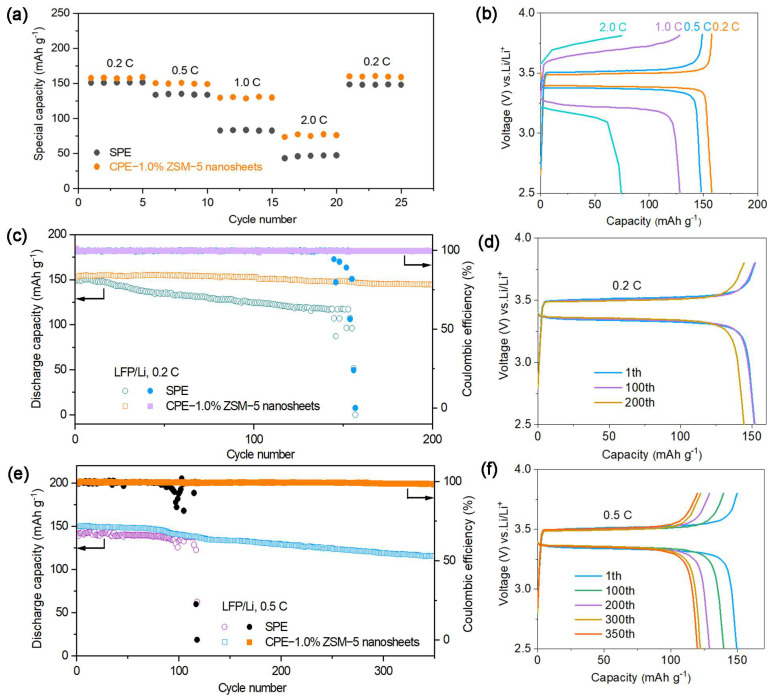
(**a**) The rate performances at different rates; (**b**) charge and discharge curves; (**c**) the cycling performance and (**d**) charge/discharge voltage curves at 0.2 C; (**e**) the cycling performance and (**f**) charge/discharge voltage curves at 0.5 C.

The rate performance of the LFP/Li cells is tested at various currents ranging from 0.2 to 2 C at 60 °C. Figure 8a presents the discharge specific capacities of a cell with CPE-1.0% ZSM-5 nanosheets are 157.8, 150.5, 130.6, and 76.9 mAh g^−1^ at rates of 0.2, 0.5, 1.0, and 2.0 C, indicating the better cyclic stability and reversibility of the CPE-1.0% ZSM-5 nanosheets than those of the pure SPE. When the current returns to 0.2 C, the discharge capacity almost recovers completely, with a coulombic efficiency of over 99%. Figure 8b represents the corresponding charge and discharge profiles of solid-state LiFePO_4_/Li cells at different rates. Therefore, the electrochemical stability of the novel CPE is promoted by the ZSM-5 nanosheets.

As illustrated in Figure 8c, the cycling performance of LFP/Li cells with CPE-1.0% ZSM-5 nanosheets attains an initial discharge capacity of 152.3 mAh g^−1^ and 91.4% capacity retention at 0.2 C after 200 cycles under 60 °C. The coulombic efficiency fluctuates around 99%. However, the cell with the pure SPE delivers a specific capacity of 51.3 mAh g^−1^ after 157 cycles. The high internal resistance and poor interfacial stability result in a rapid capacity decay. As shown in Figure 8d, an almost stable polarization voltage after 200 cycles is the same as that of the first cycle.

The cycling performance of the CPE-1.0% ZSM-5 nanosheets at 0.5 C at 60 °C (Figure 8e) and the charge–discharge curves at different cycles (Figure 8f) are checked to investigate the feasibility of the electrolytes under a high current density. A stable electrochemical performance with a discharge specific capacity of 118.8 mAh g^−1^ and a capacity retention rate of 79.6% is still observed after 350 cycles. However, the discharge specific capacity of the PEO/LiTFSI cell decays to 62.4 mAh g^−1^ after 118 cycles.

Additionally, the electrochemical stability and the discharge capacity of the cell with CPE-1.0% ZSM-5 nanosheets are higher than those of other reported PEO-based electrolytes, as listed in Table 1. The excellent compatibility with the Li electrode and interfacial stability would contribute to outstanding electrochemical performance, so solid-state LFP/Li batteries with CPE-1.0% ZSM-5 nanosheets demonstrate a satisfactory cycling stability and high discharge capacity.

## 4. Conclusions

Two-dimensional ZSM-5 nanosheets were synthesized and doped into poly(ethylene oxide) (PEO)-based electrolytes to obtain organic–inorganic composite polymer electrolytes, CPEs, for lithium metal batteries. It was confirmed that the optimal amount of the ZSM-5 nanosheets was 1.0 wt.%. The ZSM-5 nanosheet additives were effective for improving the thermal stability and mechanical properties of electrolyte membranes. The abundant silicon hydroxyls of the Lewis acid–base centers and the porous electronegativity channels offered numerous Li^+^ transfer sites. Attributed to fast lithium-ion transference, the homogeneous CPE-1.0% ZSM-5 nanosheets achieved a much higher ionic conductivity (1.34 × 10^−3^ S cm^−1^ at 60 °C), electrochemical window (4.8 V), and Li^+^ transference number (0.37) than those of the blank PEO-based electrolyte. The Li^+^ transport pathways and mechanism in the CPE-1.0% ZSM-5 nanosheets were explored. The high rigidity of the ZSM-5 nanosheets contributed to achieving inhibition of the growth of lithium dendrites for favorable interface compatibility with Li metal (over 700 h at 0.1 mA cm^−2^ without any short circuits). Moreover, the all-solid-state LiFePO_4_/Li batteries with CPEs possessed excellent cycling stabilities, with an initial discharge capacity of 152.3 mAh g^−1^ and 91.4% capacity retention after 200 cycles under 0.2 C at 60 °C. At 0.5 C, the batteries supplied 79.6% capacity retention after 350 cycles. Combined with the superior mechanical and thermal properties of the ZSM-5 nanosheets, the design and implementation in this paper could open an avenue to significantly enhance the electrochemical performance of polymer electrolytes.

## Figures and Tables

**Table 1:** Electrochemical performance of PEO-based CPEs in LFP/Li at 60 °C.

CPEs	Discharge Capacity /mAh g^−1^	Cycle Number	Operation Conditions	Capacity Retention	Ref.
LAF@LCO	122	100	0.2 C	75.1%	[25]
	/	100	0.5 C	80.2%	
ZIF-8@CMC	163.7	200	0.5 C	88.95%	[26]
LATP	151.69	100	0.5 C	85.05%	[27]
GFC	137.6	100	0.2 C	87.3%	[28]
PVDF-HFP	125.3	100	0.5 C	89.4%	[29]
2%vol Li_3_PS_4_	145.2	100	0.2 C	86.1%	[30]
	133	325	0.5 C	80.9%	
2D LDH	138	100	0.2 C	88%	[31]
5LiMPS	135.5	200	0.2 C	83.6%	[32]
15%SPVA-Li	158.4	100	0.2 C	81.5%	[33]
1.0% ZSM-5 nanosheets	152.3	200	0.2 C	91.4%	This work
	149.2	350	0.5 C	79.6%	

## Data Availability

The raw data supporting the conclusions of this article will be made available by the authors on request.
